# Consensus report from the 8th International Forum for Liver Magnetic Resonance Imaging

**DOI:** 10.1007/s00330-019-06369-4

**Published:** 2019-08-05

**Authors:** Christoph J. Zech, Ahmed Ba-Ssalamah, Thomas Berg, Hersh Chandarana, Gar-Yang Chau, Luigi Grazioli, Myeong-Jin Kim, Jeong Min Lee, Elmar M. Merkle, Takamichi Murakami, Jens Ricke, Claude B. Sirlin, Bin Song, Bachir Taouli, Kengo Yoshimitsu, Dow-Mu Koh

**Affiliations:** 1 Radiology and Nuclear Medicine, University Hospital Basel, 4031 Basel, Switzerland; 2 Department of Biomedical Imaging and Image-Guided Therapy, Medical University of Vienna, 1090 Vienna, Austria; 3 Section of Hepatology, Clinic for Neurology; Department of Internal Medicine, Neurology and Dermatology, University Hospital Leipzig, 04103 Leipzig, Germany; 4 Center for Advanced Imaging Innovation and Research (CAI2R), Department of Radiology, New York University School of Medicine, New York, NY 10016 USA; 5 Bernard and Irene Schwartz Center for Biomedical Imaging, Department of Radiology, New York University School of Medicine, New York, NY 10016 USA; 6 Division of General Surgery, Department of Surgery, Taipei Veterans General Hospital, National Yang-Ming University, Taipei, 112 Taiwan; 7 Department of Radiology, Spedali Civili di Brescia, 25123 Brescia, Italy; 8 Department of Radiology, Yonsei University College of Medicine, Seoul, 120-752 South Korea; 9 Department of Radiology, Seoul National University Hospital, Seoul, 110-744 South Korea; 10 Department of Diagnostic and Interventional Radiology, Kobe University Graduate School of Medicine, Kobe, 650-0017 Japan; 11 Klinik und Poliklinik für Radiologie, Ludwig-Maximilians-Universität München, Munich, Germany; 12 Liver Imaging Group, University of California San Diego, San Diego, CA 92093-0888 USA; 13 Department of Radiology, West China Hospital, Sichuan University, Chengdu, 610041 People’s Republic of China; 14 Department of Diagnostic, Molecular and Interventional Radiology and Translational and Molecular Imaging Institute, Icahn School of Medicine at Mount Sinai, New York, NY 10029-6574 USA; 15 Department of Radiology, Fukuoka University Faculty of Medicine, Fukuoka City, 801-1011 Japan; 16 Department of Radiology, Royal Marsden Hospital and The Institute of Cancer Research, London, SM2 5NG UK

**Keywords:** Gadoxetic acid, Hepatocellular carcinoma, Magnetic resonance imaging

## Abstract

**Objectives:**

The 8th International Forum for Liver Magnetic Resonance Imaging (MRI), held in Basel, Switzerland, in October 2017, brought together clinical and academic radiologists from around the world to discuss developments in and reach consensus on key issues in the field of gadoxetic acid–enhanced liver MRI since the previous Forum held in 2013.

**Methods:**

Two main themes in liver MRI were considered in detail at the Forum: the use of gadoxetic acid for contrast-enhanced MRI in patients with liver cirrhosis and the technical performance of gadoxetic acid–enhanced liver MRI, both opportunities and challenges. This article summarises the expert presentations and the delegate voting on consensus statements discussed at the Forum.

**Results and conclusions:**

It was concluded that gadoxetic acid–enhanced MRI has higher sensitivity for the diagnosis of hepatocellular carcinoma (HCC), when compared with multidetector CT, by utilising features of hyperenhancement in the arterial phase and hypointensity in the hepatobiliary phase (HBP). Recent HCC management guidelines recognise an increasing role for gadoxetic acid–enhanced MRI in early diagnosis and monitoring post-resection. Additional research is needed to define the role of HBP in predicting microvascular invasion, to better define washout during the transitional phase in gadoxetic acid–enhanced MRI for HCC diagnosis, and to reduce the artefacts encountered in the arterial phase. Technical developments are being directed to shortening the MRI protocol for reducing time and patient discomfort and toward utilising faster imaging and non-Cartesian free-breathing approaches that have the potential to improve multiphasic dynamic imaging.

**Key Points:**

• *Gadoxetic acid–enhanced MRI provides higher diagnostic sensitivity than CT for diagnosing HCC.*

• *Gadoxetic acid–enhanced MRI has roles in early-HCC diagnosis and monitoring post-resection response.*

• *Faster imaging and free-breathing approaches have potential to improve multiphasic dynamic imaging.*

**Electronic supplementary material:**

The online version of this article (10.1007/s00330-019-06369-4) contains supplementary material, which is available to authorized users.

## Introduction

The 8th International Forum for Liver Magnetic Resonance Imaging (MRI) was held in October 2017 in Basel, Switzerland, and attended by 119 radiologists from Asia (*n* = 46), Australia (*n* = 4), Europe (*n* = 48), North America (*n* = 20), and South America (*n* = 1). Delegates were invited to attend who had knowledge of MRI of the liver, including the use of gadoxetic acid. Two main themes were explored at the Forum: (1) the applications of gadoxetic acid (Primovist®, Eovist®) for contrast-enhanced MRI in patients with liver cirrhosis and (2) the technical performance of gadoxetic acid–enhanced liver MRI, including opportunities and challenges. As in previous Forums [1–7], consensus statements on selected topics were proposed based on the available evidence and the expert opinions of participants. The final consensus statements and the voting by delegates are presented in this article. The online supplement includes additional information on delegates’ views on current practice in liver MRI, based on a questionnaire that was circulated pre-meeting.

## Use of gadoxetic acid in patients with liver cirrhosis

### Washout in the transitional phase

**Fig. 1:**
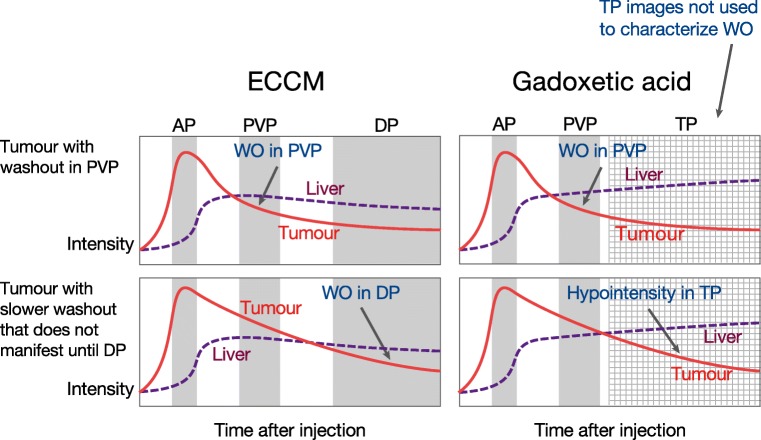
Hypoenhancement of tumour tissue relative to liver: defined as washout (WO) in the portal venous phase (PVP) or delayed phase (DP) with ECCM, but as washout in the PVP alone—not the transitional phase (TP)—with gadoxetic acid. AP, arterial phase; ECCM, extracellular contrast medium

The 2017-dated Liver Imaging Reporting and Data System (LI-RADS), updated in 2018 [8, 9], defines washout appearance on contrast-enhanced MRI as a visually assessed temporal reduction in the enhancement in tumour relative to the composite liver tissue, resulting in extracellular phase hypoenhancement. In other words, following initial enhancement in the arterial phase (AP, sometimes referred to as “washin”), “washout” is the relative hypointensity of the observation compared with background liver tissue. With extracellular contrast media (ECCM), this hypoenhancement can be assessed either in the portal venous phase (PVP) *or* the delayed phase (sometimes referred to as the “equilibrium” phase and obtained at about 3 min) *or* both. For gadoxetic acid, the hypoenhancement must be assessed only in the PVP; hypointensity in the transitional phase (TP) of gadoxetic acid—equivalent in post-injection time to the delayed phase of ECCM—does not qualify as washout. The theoretical rationale for this is that lesion hypointensity in the TP could be due to one (or both) of two things: true washout, or simply the increasing enhancement of the surrounding liver from intra-hepatocellular contrast uptake [10–13] (Fig. 1).

In contrast to LI-RADS v2018, which restricts the interpretation of washout to the PVP only in order to maintain high specificity [9], the Korean Liver Cancer Study Group (KLCSG-NCC) guidelines v2014 allow identification of washout in the PVP or the TP [14], and the Liver Cancer Study Group of Japan (LCSGJ) v2014 permits identification of washout in the PVP, TP, and even in the hepatobiliary phase (HBP) of gadoxetic acid [15]. The approach advocated by these Asian guidelines *increases the sensitivity but decreases the specificity* of the diagnosis of HCC (see below). It can also lead to incorrect interpretation of so-called pseudo-washout in non-hepatocyte-containing lesions (e.g. high-flow haemangiomas and hypervascular cholangiocarcinomas).

Four retrospective studies from South Korea assessed the specificity and sensitivity of lesion washout during the PVP alone (defined as 50–60 s after gadoxetic acid injection) or in combination with hypointensity in the TP (3 min after gadoxetic acid injection) for diagnosing HCC [16–19]. Washout in the PVP provided high specificity for diagnosis of HCC in patients with chronic liver disease, while washout in the PVP combined with hypointensity in the TP lowered the specificity (from 97.9 to 86.3% [18]), 92.9 to 78.6% [19], and 100 to 94.9% [17]). In contrast, the sensitivity for the detection of HCC increased when washout in the PVP was combined with hypointensity in the TP (from 70.9 to 86.6%, 66.7 to 72.9%, and 63.6 to 76.6%, respectively). At the time of the Liver Forum, no published data were identified on alternative timings of hypointensity assessment in the TP, e.g. 2 min after injection, and no study had been reported on the changes in specificity and sensitivity of diagnosis in the context of LI-RADS criteria, i.e. after excluding LR-1 (definitely benign), LR-2 (probably benign), and LR-M (probably or definitely malignant but not HCC-specific).

Subsequently published studies have emphasised the increased sensitivity with limited loss of specificity if hypointensity on the HBP is included for diagnosing HCC [20–23].

#### Consensus statement 1

To maximise specificity for HCC diagnosis using gadoxetic acid–enhanced MRI in patients with cirrhosis or other risk factors and based on the current literature, “washout” of gadoxetic acid should be assessed only in the PVP and not in the 3-min TP. *[72/78 (92.3%) agreement]*

#### Consensus statement 2

Further research is needed to understand the TP using gadoxetic acid. In particular, research is needed to define the physiological beginning and end of the TP based on imaging features rather than fixed time points. Relevant imaging features are likely to include the relative signal intensity of liver parenchyma in comparison to intrahepatic vessels. *[81/88 (92.1%) agreement]*

#### Consensus statement 3

Further research is needed to define when post-AP hypointensity can no longer be interpreted as “washout” with gadoxetic acid–enhanced MRI. Because the temporal signal intensity of gadoxetic acid–enhanced MRI varies across individuals, imaging features (as informed by Consensus statement 2) should be used in this definition, if possible, instead of fixed time points. *[81/88 (92.1%) agreement]*

### Specificity of the hepatobiliary phase to diagnose HCC

Kim et al [24] reported that the detection sensitivity for high-grade dysplastic nodules (HGDN), early HCC, and progressed HCC was significantly higher with gadoxetic acid–enhanced MRI than with multidetector computed tomography (CT). Most HGDNs (82.4%) and early HCCs (76.2%) demonstrated hypointensity on HBP images.

Hyperenhancement in the AP and washout are described as the most specific imaging features for the diagnosis of HCC in gadoxetic acid–enhanced MRI [25]. Bartolozzi et al reported that lesion enhancement on vascular dynamic and HBP imaging significantly correlated with the histological diagnosis of HCC (*p* < 0.0001) [25]. Golfieri et al found that HBP hypointensity by itself was the strongest MRI marker of malignancy in atypical cirrhotic nodules in gadoxetic acid–enhanced MRI; HBP hypointensity alone had 88% sensitivity, 97% specificity, 91% negative predictive value, and 93% diagnostic accuracy, significantly superior to any other MRI feature alone or combined [26]. Yoon et al described in a retrospective study of gadoxetic acid–enhanced MRI that a significant proportion of non-hypervascular HBP hypointense nodules (≥ 1 cm in diameter) in patients with cirrhosis showed malignant features on pathology (73.1%) [27]. In another group of patients whose hypointense HBP nodules were followed up for at least 12 months (mean 19 ± 10 months), 32.7% of nodules developed hypervascularity and 78.8% showed at least one imaging feature considered to indicate malignant change [27]. However, hypointensity in the HBP by itself cannot differentiate HGDN from early or progressed HCC.

Recommendations on terminology for nodules without AP hyperenhancement and with HBP hypointensity in chronic liver disease have subsequently been published by Motosugi et al on behalf of the LI-RADS HBA Working Group [28].

#### Consensus statement 4

Hypointensity in the HBP of gadoxetic acid–enhanced MRI is the most sensitive imaging feature for the diagnosis of an HGDN, an early HCC, or a progressed HCC in a patient at risk for HCC. *Notes:* (1) This statement applies regardless of vascularity in the AP; (2) hypointensity by itself has other potential diagnoses (e.g. haemangiomas), besides HCC. *[74/89 (83.2%) agreement]*

#### Consensus statement 5

Using multiparametric MRI (including T2-weighted [T2W] and diffusion-weighted imaging [DWI]), high specificity (> 90%) for the diagnosis of HCC can be achieved when hypointensity in the HBP is associated with solid arterial enhancement, regardless of venous washout, in patients with liver cirrhosis. *Note:* A small percentage of such lesions may represent other entities, e.g. cholangiocarcinoma or combined HCC-cholangiocarcinoma, while cysts and haemangiomas can be ruled out by the signal in T2W and DWI sequences. *[74/87 (85.1%) agreement]*

#### Consensus statement 6

As a significant number of hypovascular nodules (> 1 cm) that are hypointense on HBP are, or eventually become (by 6–12 months), HCC in patients with liver cirrhosis, close observation or intervention is recommended. *Note:* The current statement reflects practice differences around the world, including the availability of surgery, ablation, and biopsy. *[80/88 (90.9%) agreement]*

### Detection of venous invasion with gadoxetic acid–enhanced MRI

Macrovascular invasion is a characteristic feature of advanced HCC, indicating high risk for metastasis, recurrence, liver functional impairment, and poor prognosis (Barcelona Clinic Liver Cancer [BCLC] stage C) [29]. Microvascular invasion is increasingly recognised as a risk factor for intrahepatic metastases in BCLC stage B/A HCC patients, and even stage 0 HCC patients [30, 31].

Certain imaging features on gadoxetic acid–enhanced MRI in the HBP have been shown in retrospective studies to be predictive for microvascular invasion with high specificity, including the presence and degree of peritumoural hypointensity [13, 32, 33], peritumoural-decreased uptake area [33], and an irregular and non-smooth tumour margin [34]. The presence of intratumoural fat correlated negatively with microvascular invasion, suggesting intratumoural fat may indicate a lower risk for microvascular invasion [35]. However, these findings need confirmation in independent studies.

#### Consensus statement 7

In HCC patients, retrospective single-centre studies suggest that peritumoural HBP hypointensity may predict the presence of microvascular invasion. Prospective multicentre studies are needed to validate peritumoural HBP phase hypointensity for predicting microvascular invasion in HCC, especially in those with HCC < 2 cm. *[39/43 (90.7%) agreement]*

### Differential diagnosis of HCC

The key imaging findings of HCC (1–2 cm) in LI-RADS v2018 are arterial phase hyperenhancement (APHE, washin), PVP or delayed phase washout, capsular appearance, and threshold growth [8]. These criteria are based on dynamic CT or on MRI with either ECCM (PVP or delayed washout) or gadoxetic acid (PVP washout only) [8]. ECCM and gadoxetic acid are both gadolinium-based contrast agents, but they are not interchangeable because of the differences in pharmacokinetics, dosage, and mechanism of action [36].

Two meta-analyses showed superior sensitivity and positive predictive value (PPV) or negative likelihood ratio for gadoxetic acid–enhanced MRI compared with CT for HCC detection [37, 38]. One of the meta-analyses reported equivalent sensitivity of gadoxetic acid and ECCM for HCC detection [38]. However, gadoxetic acid–enhanced MRI, compared with CT and ECCM-based MRI, may provide alternative imaging features that help differentiate classic HCC from non-HCC malignant lesions. One of the most important features of gadoxetic acid–enhanced MRI is the targetoid HBP pattern—i.e. central hyperintensity with peripheral hypointensity in the rim in the HBP—which can assist to differentiate non-HCC malignant lesions (combined HCC-cholangiocarcinoma, intrahepatic cholangiocarcinoma) from HCC; see Consensus statement 9 [8, 39–41].

#### Consensus statement 8

There is enough evidence (level 2) to recommend gadoxetic acid–enhanced MRI over contrast-enhanced CT as the primary imaging modality for imaging HCC worldwide. There is not enough evidence currently to support a similar statement comparing gadoxetic acid–enhanced MRI against MRI using ECCM. *[60/72 (83.3%) agreement]*

#### Consensus statement 9

Compared with CT or ECCM-based MRI, gadoxetic acid–enhanced MRI may provide alternative imaging features that help in differentiating classic HCC from non-HCC malignant lesions (combined HCC-cholangiocarcinoma, intrahepatic cholangiocarcinoma). One of the most important such features is the targetoid HBP pattern, which increases the likelihood of a non-HCC malignancy and by itself suffices for assigning an LR-M category using LI-RADS. *Notes:* (1) Due to fibrous or desmoplastic components, the targetoid pattern can be seen in some HCC and rare benign lesions, e.g. sclerosed haemangiomas; (2) combined tumours and small cholangiocarcinomas may overlap in imaging appearance and can be difficult to differentiate from HCC. *[66/83 (79.5%) agreement]*

#### Consensus statement 10

Comparative studies have demonstrated that gadoxetic acid–enhanced MRI provides higher sensitivity with similar specificity for detecting malignancy when compared with contrast-enhanced CT in cirrhotic liver. Prospective studies are needed to compare the diagnostic performance of gadoxetic acid–enhanced MRI with ECCM. *[67/83 (80.7%) agreement]*

### Capsule appearance in HCC with gadoxetic acid–enhanced MRI

An enhancing “capsule” belongs to the major features of HCC in the LI-RADS v2018 guidelines, where it is defined as a peripheral rim of smooth hyperenhancement in the PVP, DP, or TP [8]. The conventional capsule appearance is a useful feature for diagnosing HCC [42] that has been investigated in a comparative study of gadoxetic acid and gadobenate dimeglumine [43]. However, the capsule appearance may be obscured by the rapid disappearance of contrast medium from the blood pool and by hyperenhancement of the background liver on gadoxetic acid–enhanced MRI, which are associated with inconsistent sensitivities reported to range from around 20 to 90% [44–47].

A hypointense rim on the HBP phase is considered an ancillary feature in the LI-RADS v2018 guidelines and has the potential to improve detection of the capsule, favouring the diagnosis of HCC in addition to the conventional capsule appearance alone.

Incorporating the assessment of a hypointense rim in the HBP phase significantly improves the sensitivity and accuracy for the detection of a histological capsule compared with conventional capsule assessment (81.5 vs. 57.8% and 76.1 vs. 59.4%, respectively; *p* < 0.001) [48]. Combined assessment of the HCC capsule using the conventional technique and the hypointense rim during the HBP can significantly improve the sensitivity and accuracy for the diagnosis of HCC compared with conventional capsule assessment alone (83.0 vs. 72.7% and 84.1 vs. 75.1%, respectively; *p* < 0.001), with the same specificity (91.5%) [48].

#### Consensus statement 11

The conventional capsule appearance, a major feature in LI-RADS, may be obscured by the rapid disappearance of contrast medium from the blood pool when using ECCM or gadoxetic acid and progressive hyperenhancement of the background liver on gadoxetic acid–enhanced MRI, resulting in inconsistent sensitivities. *[73/82 (89.0%) agreement]*

#### Consensus statement 12

A smooth hypointense rim on the HBP phase, currently considered an ancillary feature in LI-RADS, may have the potential to be included as a capsule appearance. *[66/83 (79.5%) agreement]*

### Detection of HCC foci prior to therapy decisions

Curative treatment options—including resection, liver transplantation, and locoregional therapy such as radiofrequency ablation (RFA)—are applicable only for early-stage HCC conforming to the Milan Criteria [49, 50]. Precise staging is therefore key to the optimal management of HCC.

**Fig. 2:**
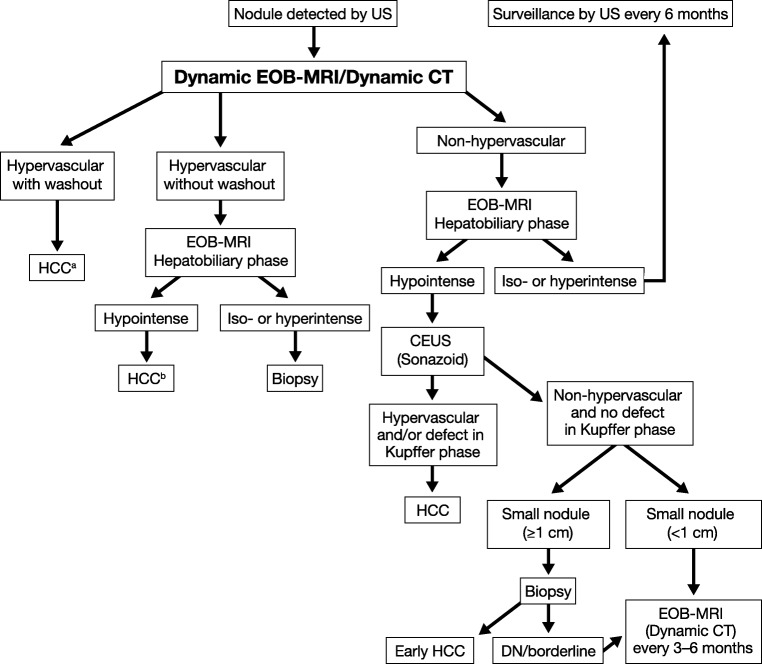
Asian Pacific Association for the Study of the Liver (APASL) Guidelines 2017 [52]. From Omata M, et al Hepatol Int. 11:317–370, with permission. ^a^Cavernous haemangioma sometimes shows hypointensity on the equilibrium (transitional) phase of dynamic Gd-EOB-DTPA MRI (pseudo-washout). It should be excluded by further MRI sequences and/or other imaging modalities. ^b^Cavernous haemangioma usually shows hypointensity on the hepatobiliary phase of Gd-EOB-DTPA MRI. It should be excluded by other MRI sequences and/or other imaging modalities. CT, computed tomography; DN, dysplastic nodule; Gd-EOB-DTPA, gadoxetic acid; HCC, hepatocellular carcinoma; MRI, magnetic resonance imaging; US, ultrasound

In the more recent versions of management guidelines, there has been an increasing role for hepatobiliary contrast-enhanced MRI, greater use of follow-up imaging instead of biopsy, and recommendation for a single dynamic study (either CT or MRI) rather than two dynamic imaging modalities for the diagnosis of small-diameter (< 2 cm) HCC [8, 51, 52]. For example, in the Asian Pacific Association for the Study of the Liver (APASL) guideline [52], the diagnostic algorithm is based on dynamic patterns and gadoxetic acid–enhanced MRI is included as a first-line diagnostic tool for HCC (Fig. 2).

Geographical differences exist between guidelines, driven largely by differences in treatment practices. In North America and Europe, the greatest concern is for high specificity. Since patients with a diagnosis of HCC may undergo liver transplantation based on imaging criteria alone, stringent diagnostic criteria are used to avoid false-positive HCC diagnoses [8, 29]. In the European Association for Study of Liver-European Organisation for Research and Treatment of Cancer (EASL-EORTC) guidelines, the radiological hallmark for HCC is APHE and portal venous/delayed phase washout [29]. In the 2017 American Association for the Study of Liver Diseases (AASLD) guidelines, MRI with ECCM or gadoxetic acid is concluded to provide higher pooled sensitivity and similar specificity compared with CT [51]. However, the AASLD still recommends diagnostic evaluation of HCC with *either* multiphasic CT *or* multiphasic MRI because they assume a similar diagnostic performance. While this statement is strongly endorsed by the AASLD guidelines, the supportive evidence level for CT is relatively low. There are no randomised, controlled trials comparing CT, ECCM-enhanced MRI, and gadoxetic acid–enhanced MRI for the diagnosis of HCC in cirrhotic patients.

In contrast to the approach in North America and Europe, in Asia, the primary aim is to maximise the sensitivity of HCC diagnosis. This is justified by the greater use of locoregional ablative therapies in Asia such as percutaneous ethanol injection, RFA, and transarterial chemoembolisation. In the APASL guidelines, gadoxetic acid–enhanced MRI is preferred over ECCM-enhanced MRI as a first-line diagnostic test. Hypointensity on HBP imaging can replace washout on ECCM MRI. Typical HCC can be diagnosed by imaging, regardless of its size, by applying the “washin/washout criteria” [52].

A recent meta-analysis concluded that CT and ECCM-based MRI show similar diagnostic performance for detecting HCC, while gadoxetic acid–enhanced MRI has the highest overall sensitivity and PPV and may be the optimal single method for diagnosis of HCC [37]. Another meta-analysis concluded that MRI with an ECCM or gadoxetic acid has a significantly higher sensitivity (82% vs. 66%) and lower negative likelihood ratio (0.20 vs. 0.37) versus CT, with no differences between the techniques in specificities and positive likelihood ratios [38].

A retrospective comparison against dynamic CT for staging HCC reported that gadoxetic acid–enhanced MRI provided significantly greater sensitivity (90.6% vs. 79.5%; *p* < 0.0001) and more accurate BCLC staging (92.8% vs. 80.5%; *p* < 0.0001). BCLC stage was correctly changed after gadoxetic acid–enhanced MRI in 13.8% patients [53]. Gadoxetic acid–enhanced MRI was also superior to CT for detecting intrahepatic recurrence post-curative surgery in patients with HCC. In a lesion-by-lesion analysis, the sensitivity was significantly higher for gadoxetic acid–enhanced MRI than on multidetector CT (*p* < 0.005, both study reviewers) [54].

#### Consensus statement 13

Gadoxetic acid–enhanced MRI is an accurate method for the diagnosis and staging of HCC (level 2 evidence in meta-analyses). Although the reported accuracy of gadoxetic acid–enhanced MRI for diagnosis of HCC compares favourably with that of CT or ECCM-enhanced MRI, the quality of the evidence is still insufficient to recommend gadoxetic acid–enhanced MRI over CT or ECCM-enhanced MRI in all patient populations. *Notes:* (1) The statement “in all patient populations” relates to different management patterns in different regions and aims to capture differences in Western versus Asian treatment practices (mentioned above). (2) “Gadoxetic acid–enhanced MRI” refers to the entire package of imaging and not simply to the HBP. *[65/87 (74.7%) agreement]*

#### Consensus statement 14

Gadoxetic acid–enhanced MRI is useful for the preoperative staging of HCC, and also for follow-up after surgery of HCC, as it can detect new lesions with high sensitivity. *[82/88 (93.2%) agreement]*

## Technical-related issues

### Artefacts in the arterial phase

Arterial phase (AP) imaging is an essential component in gadoxetic acid–enhanced MRI for making an HCC diagnosis and monitoring treatment response, identifying AP hyperenhancing benign lesions and liver metastases, and assessing hepatic artery anatomy. Artefacts have been described in the literature that may influence image quality in the AP.

The first description of “acute transient dyspnoea” after administration of gadoxetic acid was published in 2013 [55]. In this single-centre prospective, nonrandomised observational study, 198 patients underwent MRI of the abdomen (99 with gadoxetic acid, 99 with gadobenate dimeglumine). A proportion of patients—14% in the gadoxetic acid and 5% in the gadobenate dimeglumine group (*p* = 0.05)—described a temporary, self-limiting phenomenon lasting for 10–20 s, during which they felt as if they “couldn’t catch their breath”. Concomitantly, there were cases of image quality being severely degraded by patient respiratory motion during the AP, which were more frequent in the gadoxetic acid than in the gadobenate dimeglumine group, both for all patients (17 vs. 2%, *p* = 0.0007) and for the cirrhotic subpopulation (19 vs. 3%, *p* = 0.02). This effect did not extend to PVP, transitional phase, or HBP.

A follow-up retrospective study by the same group [56] on 180 patients who underwent gadoxetic acid– and gadobenate dimeglumine–enhanced MRI at different times reported a higher incidence of respiratory motion-related artefact in the AP (transient severe motion [TSM] artefact) associated with gadoxetic acid than gadobenate (39% vs. 10%, *p* < 0.0001; severe in 18% vs. 2%, respectively, *p* < 0.0001).

In a recent review of the published literature, the prevalence of TSM associated with gadoxetic acid–enhanced MRI of the liver ranged from 5 to 22% [57]. There are major geographical differences in the prevalence of TSM, with higher rates in patients in the USA compared with those in Asia [58].

The mechanism underlying TSM is unclear. Reported risk factors for TSM in gadoxetic acid–enhanced MRI have included male sex, high body mass index, breath-hold failure [58, 59], presence of chronic obstructive pulmonary disease [59], higher (off-label) gadoxetic acid injection doses [59], and history of prior TSM [60, 61].

Proposed solutions to TSM are also widely debated, without current consensus [53–69]. In a recent prospective observational study in 250 consecutive patients, the incidence of acute transient dyspnoea after gadoxetic acid administration was reported in less than 1% and combination with a multiarterial phase technique significantly reduced the incidence of artefacts [62].

#### Consensus statement 15

Gadoxetic acid–enhanced MRI has been associated in the literature with artefacts during the AP that are suspected to be secondary to TSM, as recently described. TSM has an estimated prevalence of 2–39% (mean 15%). The mechanism of TSM is unclear. *[71/76 (93.4%) agreement]*

#### Consensus statement 16

Several possible solutions have been suggested to minimise the artefact of TSM. These include the use of multiple APs, shortening the acquisition, use of Cartesian and non-Cartesian free-breathing acquisition, contrast dilution, and changes in timing methods. Further research and a consensus are needed to determine which method should be proposed to decrease/eliminate this artefact. [*87/89 (97.8%) agreement]*

### Shortened MRI protocols

**Fig. 3:**
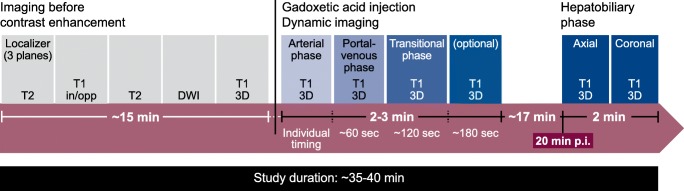
Traditional MRI protocol using gadoxetic acid—T1W, T2W, and DWI are performed before gadoxetic acid administration. DWI, diffusion-weighted imaging

A major objective in protocol optimisation is to provide high-quality images within as short a time as possible. Discussion of the optimal protocol for gadoxetic acid–enhanced MRI was included in the First Liver Forum Consensus Manuscript 11 years ago [1]. In the “traditional” protocol (Fig. 3), non-contrast sequences are acquired before gadoxetic acid injection, followed by dynamic evaluation. After a wait of 10 min in patients with normal liver function and 20 min in patients with liver cirrhosis or otherwise compromised liver function, the HBP images are acquired. The total protocol duration is approximately 35–40 min.

**Fig. 4:**
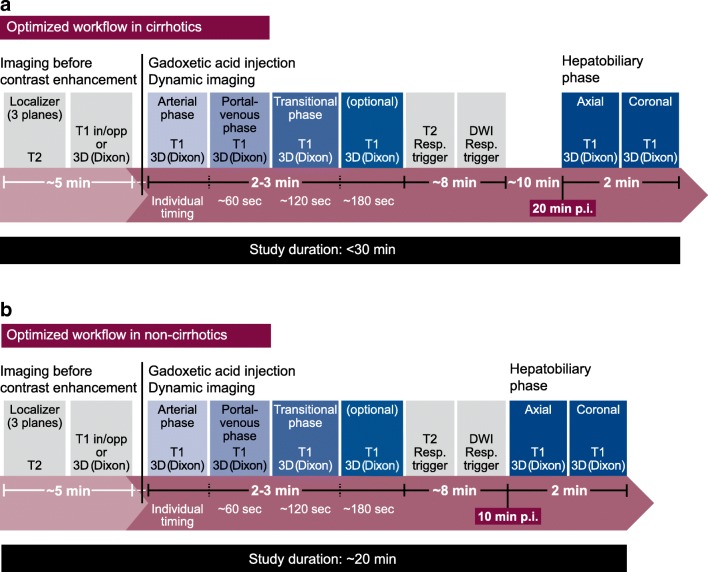
Optimised MRI protocol using gadoxetic acid. DWI, diffusion-weighted imaging

In an alternative “optimised” protocol using gadoxetic acid (Fig. 4), pre-contrast T2W and DWI are moved before the HBP. This can save 5 or even 8 min in the protocol. The time saved using the optimised protocol can represent a decrease in patient discomfort and costs relative to the traditional protocol or provide an opportunity to perform optional additional acquisitions.

Studies have demonstrated that if T2W and DW images are acquired after gadoxetic acid injection in the optimised protocol, the same results and comparable diagnostic capability are obtained as in the “traditional” protocol [63, 64]. An exception to the recommendation to use the optimised protocol is suspected haemangioma, where it is suggested to perform T2 and DWI before gadoxetic acid injection due to the high specificity of T2W MRI in this situation [65, 66]. The results can inform whether there is subsequently a need to inject gadoxetic acid. Moreover, a T2W-based MR cholangiography cannot be performed after gadoxetic acid injection, unless it is acquired within 5 min after gadoxetic acid injection.

Additional steps to consider when using an optimised MRI protocol include (1) using bolus timing techniques for optimal enhancement during the dynamic phase [67] and (2) starting the HBP at about 10 min post-injection to save examination time. Although a 20-min delay can improve the signal intensity and liver-to-lesion contrast with benefit in some situations, about a 10-min delay is sufficient in most patients without chronic liver disease [68]. (3) High flip angle delayed HBP imaging is a useful adjunct to standard-enhanced MRI of the liver. It allows for better sensitivity in focal liver detection, particularly for small lesions, and this technique increases the conspicuity of the biliary system, which is an additional benefit of delayed imaging [69–71].

#### Consensus statement 17

The minimum protocol in the cirrhotic/high-risk HCC-patient for screening and pre-surgical evaluation/staging incudes:

- T1W in-phase and out-of-phase
- Pre-contrast plus dynamic contrast-enhanced evaluation
- T2W and DWI acquisition
- HBP at 20 min
- Optional additional sequences can be used for specific clinical situations or according to institutional preference *[71/83 (85.5%) agreement]*

#### Consensus statement 18

The minimum protocol in the non-cirrhotic oncological patient (pre- and post-treatment) consists of:

- T1W in-phase and out-of-phase
- Pre- and post-contrast contrast-enhanced dynamic evaluation
- T2W and DWI acquisition
- HBP at 10–15 min
- Optional additional sequences can be used for specific clinical situations or according to institutional preference
- *Notes:* In biliary-related diseases, perform T2 (and DWI) and MR cholangiography before contrast administration. Prospective studies are needed to see if the HBP alone may suffice in the follow-up setting *[70/85 (82.4%) agreement]*

An additional protocol suggestion to use gadoxetic acid–enhanced MRI when finding an incidental lesion in a healthy patient did not reach overall consensus (> 50% agreement) and is not included among the consensus statements.

### New sequences for dynamic imaging—current clinical value

There are current limitations to dynamic contrast-enhanced MRI of liver lesions, including (1) the requirement for an AP and PVP that show an arterial phase–enhancing lesion and washout compatible with HCC and (2) the need for spatial resolution sufficient to detect 1–2-cm tumours combined with temporal resolution sufficient to visualise contrast dynamics within a single breath hold. Additional factors when using gadoxetic acid are an injection rate 1 cc/s related to a smaller injection volume and a lower total gadolinium dose (usually 0.025 mmol Gd/kg BW) compared with other ECCM, no prior timing run, and concerns over respiratory motion-related artefacts.

Solutions to these limitations are being developed. One solution is faster imaging, during a single breath hold, achieved by (1) parallel imaging, through undersampling of the κ-space, and use of receiver coils that provide spatial information to unwrap the image [72]; and (2) view-sharing, utilising keyhole imaging techniques, e.g. TRICKS (time-resolved imaging of contrast kinetics) and TWIST (time-resolved angiography with stochastic trajectories), to provide more frequent sampling of the centre of the κ-space compared with the periphery. In one study using gadoxetic acid, Pietryga et al used a high parallel acceleration factor to perform multiple APs in one breath hold. The technique recovered most APs that would otherwise have been compromised by transient motion [73].

A second solution is to use a free-breathing approach. Among multiple potential approaches, one is to acquire data in a STAR VIBE (radial volumetric interpolated breath-hold examination) sequence [74]. Fat-suppressed STAR VIBE is acquired with stack-of-stars κ-space sampling, which uses conventional sampling in the slice direction and radial sampling in-plane. To speed up radial acquisition, compressed sensing can be used, a method that exploits the compressibility or sparsity of MRI data to reconstruct under-sampled data. This is the concept underlying the GRASP (golden-angle radial sparse parallel) technique, which combines a golden-angle ordering scheme, compressed-sensing reconstruction, and parallel imaging.

The technique acquires continuous data over approximately 3–5 min in a radial fashion while the patient breathes normally. All required phases can then be reconstructed retrospectively, so that multiple APs and venous phases can be obtained together. A study using this technique to extract perfusion parameters found—as expected—that total plasma flow was reduced but additionally showed that the hepatocellular uptake rate for gadoxetic acid was lower in cirrhotic compared with that in non-cirrhotic liver [75].

XD-GRASP represents a further development in GRASP imaging, utilising retrospective motion-resolved reconstruction for image acquisition, spanning from end-inspiration to end-expiration, to acquire images. Chandarana et al reported that free-breathing, motion-resolved XD-GRASP reconstructions provide high-quality multiphase images in patients undergoing gadoxetic acid–enhanced liver MRI, superior in image quality to standard GRASP reconstructions [76]. Other techniques that enable rapid robust imaging are being investigated and have the potential to help improve the image quality of contrast-enhanced liver exams. Dedicated clinical studies will be required to demonstrate the value of these novel techniques.

#### Consensus statement 19

Conventional contrast-enhanced multiphasic MRI remains limited with the need for (1) higher spatial resolution (that more closely resembles CT) in the AP as well as higher temporal resolution to shorten the breath hold and (2) greater robustness to eliminate/minimise motion-related artefacts. *[67/72 (93.1%) agreement]*

#### Consensus statement 20

Recent advances in MRI that enable fast imaging (advanced parallel imaging, view sharing, compressed sensing) and more robust imaging (non-Cartesian imaging) have the potential to improve multiphasic dynamic liver imaging in patients undergoing gadoxetic acid–enhanced liver MRI. However, more technical developments and definitive clinical studies/trials are needed to demonstrate clinical value. *[67/68 (98.5%) agreement]*

## Summary

Gadoxetic acid–enhanced MRI has an important role in the care of patients, offering a unique combination of sensitivity and specificity that is recognised in recent management guidelines, and recommended over all other contrast agents and imaging modalities in the Asian guidelines. Optimisation of the acquisition techniques, timing, and other parameters of the arterial, venous, and hepatobiliary phases is an area of ongoing research to further enhance the utility of gadoxetic acid–enhanced MRI, while other research is exploring methods to reduce associated artefacts and to utilise the latest advances in multiphasic dynamic image acquisition.

## Electronic supplementary material

ESM 1 (DOCX 75 kb)

## Abbreviations

AASLD: American Association for the Study of Liver Diseases
AP: Arterial phase
APASL: Asian Pacific Association for the Study of the Liver
APHE: Arterial phase hyperenhancement
BCLC: Barcelona Clinic Liver Cancer
BW: Body weight
CT: Computed tomography
DN: Dysplastic nodule
DP: Delayed phase
DWI: Diffusion-weighted imaging
EASL-EORTC: European Association for Study of Liver-European Organisation for Research and Treatment of Cancer
ECCM: Extracellular contrast media
EOB: Gadoxetic acid
GD: Gadolinium
Gd-EOB-DTPA: Gadoxetic acid
HBP: Hepatobiliary phase
HCC: Hepatocellular carcinoma
HGDN: High-grade dysplastic nodule
KLCSG-NCC: Korean Liver Cancer Study Group
LCSGJ: Liver Cancer Study Group of Japan
LI-RADS: Liver Imaging Reporting and Data System
MRI: Magnetic resonance imaging
PVP: Portal venous phase
RFA: Radiofrequency ablation
T2W: T2-weighted
TP: Transitional phase
TRICKS: Time-resolved imaging of contrast kinetics
TSM: Transient severe motion
TWIST: Time-resolved angiography with stochastic trajectories
US: Ultrasound
WO: Washout
