# Supplementary material for: Consensus report from the 8th International Forum for Liver Magnetic Resonance Imaging
Source: Eur Radiol. 2019 Aug 5;30(1):370–82. doi: 10.1007/s00330-019-06369-4 (PMC6890618; doi:10.1007/s00330-019-06369-4)
Supplement: Supplementary file 1 — (DOCX 75 kb) [file 330_2019_6369_MOESM1_ESM.docx]

**Supplement: Delegate responses to the pre-meeting questionnaire**

**Questions 1–3 Ask About Your Liver Imaging Practice**

**1. How do you describe your practice?**

**2. How many years of experience do you have in liver imaging?**

**3. How many years of experience do you have with gadoxetic acid?**

**Questions 4–8 Relate to Gadoxetic Acid-Enhanced MRI Technique**

**4. Which of the following technical advances have you implemented in your practice for gadoxetic acid-enhanced MRI [1–4]?**

**Question 4 footnotes:**

**CAIPIRINHA VIBE (Siemens)**

1. Park YS, Lee CH, Kim IS et al (2014) Usefulness of controlled aliasing in parallel imaging results in higher acceleration in gadoxetic acid-enhanced liver magnetic resonance imaging to clarify the hepatic arterial phase. Invest Radiol 49:183–188.

**DISCO (GE Healthcare)**

2. Saranathan M, Rettmann DW, Hargreaves BA, Clarke SE, Vasanawala SS (2012) DIfferential subsampling with cartesian ordering (DISCO): a high spatio-temporal resolution Dixon imaging sequence for multiphasic contrast enhanced abdominal imaging. J Magn Reson Imaging 35:1484–1492

**Radial VIBE (Siemens)**

3. Chandarana H, Block KT, Winfeld MJ, et al (2014) Free-breathing contrast-enhanced T1-weighted gradient-echo imaging with radial k-space sampling for paediatric abdominopelvic MRI. *Eur Radiol* 24:320–326.

**GRASP (Siemens)**

4. Feng L, Grimm R, Block KT, et al (2014) Golden-angle radial sparse parallel MRI: combination of compressed sensing, parallel imaging, and golden-angle radial sampling for fast and flexible dynamic volumetric MRI. Magn Reson Med 72:707–717.

**5. How do you time your arterial phase?**

**6. What dose do you use?**

**7. Do you dilute?**

**8. What is your usual injection rate?**

**Questions 9–14 Relate to Adult Patients with Liver Cirrhosis**

**9. Hypointensity at 2 min can be interpreted as washout**

**10. Hypointensity in the hepatobiliary phase can be interpreted as a specific feature of hepatocellular carcinoma**

**11. Compared with extracellular contrast-enhanced (ECCM) MRI, gadoxetic acid MRI permits detection of macrovascular invasion into portal and/or hepatic veins with equal accuracy and confidence**

**12. Compared with ECCM MRI, gadoxetic acid MRI permits differentiation of HCC from cholangiocarcinoma with equal accuracy and confidence**

**13. Compared with ECCM MRI, gadoxetic acid MRI permits visualization of the HCC capsule with equal accuracy and confidence**

**14. Gadoxetic acid MRI is the imaging modality with very high sensitivity for local staging of HCC prior to planned surgical resection**

**Questions 15–19 Relate to General Use of Gadoxetic Acid in Liver Imaging**

**15. In what proportion of exams do you see unequivocal degradation of image quality in the arterial phase that impairs diagnostic accuracy?**

**16. The benefits outweigh the disadvantages when performing DWI and T2 post-gadoxetic acid injection to reduce exam time**

**17. Robust motion-insensitive multiple arterial phase acquisition techniques improve diagnostic accuracy and confidence in gadoxetic acid MR**

**18. Have recent discussions on gadolinium presence in the body/brain altered your use of gadoxetic acid MRI?**

**19. Is your overall experience with the use of gadoxetic acid reflected in the questions?**
